# Geometry Optimization of the Runner Insert for Improving the Appearance Quality of the Injection Molded Auto Part

**DOI:** 10.3390/polym14194085

**Published:** 2022-09-29

**Authors:** Zhiguo Ma, Zheng Li, Ming Huang, Chuntai Liu, Changyu Shen, Xinyu Wang

**Affiliations:** 1 National Engineering Research Center for Advanced Polymer Processing Technology, Zhengzhou University, Zhengzhou 450002, China; 2 Department of Engineering Mechanics, Dalian University of Technology, Dalian 116024, China; 3 The William G. Lowrie Department of Chemical and Biomolecular Engineering, The Ohio State University, Columbus, OH 43210, USA

**Keywords:** appearance quality, deformation, geometry optimization, injection molding, insert

## Abstract

Appearance quality is one of the most important indexes for many injection-molded products, like optical parts, automotive parts etc., especially at the area near the injection gate. Different from the work that focused on designing the dimensions of the runner, this work proposed a method which is based on the insert technology to improve the appearance quality of a standard automotive part. An insert was introduced into the runner system which located before the gate. Three different shapes of this insert (circular, rectangular and diamond) were used to study the effect of geometrical factors on the appearance quality in this paper. All inserts were parameterized to describe their location and dimensions. Based on the geometrical design parameters, expected improvement optimization problem about the appearance quality were solved by using sequential approximate optimization method. The appearance qualities of three cases are improved by 13.77%, 21.56%, 14.37% respectively. Results showed that the best geometrical design scheme of the insert is rectangular with the optimal geometrical location and dimensions. The reasons were discussed by investigating the flow and thermal history in detail. Compared with the design case without any insert, the heat was absorbed and the velocity field was changed by the insert before the polymer melts ran into the cavity. It changed the complicated thermo-mechanical history inside the material during the entire processing history, which improved the final appearance quality of this auto part.

## 1. Introduction

Thermoplastic injection molding is a powerful technology in modern manufacturing industry. It has been widely used due to its outstanding advantages, like high efficiency, high precision and repeatability, low cost etc. However, there exist many processing defects, like warpage, shrinkage, weld line, sink mark etc., in injection molded parts since thermo-mechanics history of thermoplastic material is complicated. Therefore, one of the important things before mass-produce is how to avoid or reduce the defects of injection molded parts. Engineers had strong demand of good and low-cost tools to assist them to rapidly design mold and process plastic product with high quality. As computer aided engineering (CAE) technology rapidly developed, many simulation software (Moldflow, Modex3D etc.) were explored to solve the complicated physical fields and predict molding defects. Now, they have played very important role in mold and processing parameter design.

Deformation (warpage and shrinkage) exists in all injection molded plastic parts. For thin-wall parts, warpage is one of the most familiar manufacturing defects. It is necessary to measure warpage [1,2] precisely and minimize it as much as possible in injection industry. The interest of some researchers focuses on processing optimization and design. Combining CAE technology and certain mathematic knowledge, researchers have done many good works on developing intelligent methods to improve the processing quality of different products, especially by using different machine learning methods. Kitayama et al. [3] designed and optimized the packing pressure curve to reduce the warpage of the product based on sequential approximate optimization (SAO). Based on kriging surrogate model and EI function, Gao et al. [4,5] and Wang et al. [6,7] optimized processing parameters of conventional injection molding and dynamic filling and packing curve respectively to reduce the warpage. Shi et al. [8,9,10], Song et al. [11] and Bensingh et al. [12] used an ANN model-based method to improve the quality of injection molded product. Machine learning was employed by Hwang and Kim [13] to predictive warpage of a radiator tank, then combined with hybrid metaheuristic algorithm to reduce this defect. The work of Studer and Ehrig [14] was different from processing parameters optimization, thickness distribution was only taken into consideration to improve the quality of the part.

Geometric design of cooling channel, runner or gate is another factor to affect the thermo-mechanical history during processing parts. Therefore, more and more work focus on coupled parameters optimization including processing parameters and mold design parameters [15]. For instance, to minimize the warpage of the product, Ozcelik and Erzurumlu [16] employed artificial neural network (ANN)-and genetic algorithm (GA)-based optimization methods to optimized key processing parameters and two parameters about cooling channel. Wu et al. considered design parameters of runner system and processing parameters to reduce the warpage of a digital photo frame with weld line constraints. Similar, Kang et al. [17] selected fifteen parameters including temperature, packing curve and film gate, and optimized the warpage by using micro genetic algorithm.

There are some studies which combined warpage with other non-deformation index, like weld line, cycle time or clamp force etc. [18]. Shrinkage is one of them which are concerned by researchers. For instance, Abdul et al. [19] studied how to use Taguchi and ANN to predict the shrinkage of plastic part. Wu et al. [20] did their effort to investigate the effect of microscopic structures on the shrinkage of polymethyl-methacrylate (PMMA) flat specimen. However, main work was to study how to minimize warpage and shrinkage simultaneously in many literatures [21,22]. Farshi et al. [23] targeted warpage and volumetric shrinkage together of the automotive venti duct, and conducted an optimization work by using the sequential variable-size simplex algorithm. Zhou and Turng [24] developed Gaussian process surrogate model-based method to solve the process parameters optimization problem about warpage and volumetric shrinkage. Zhao et al. [25] also studied and solved the multi-objective optimization problem including warpage, shrinkage and sink marks.

Recently, the concept of conformal cooling channel has attracted more and more attention [26,27,28,29,30,31]. It can control the quality of injection-molded part by controlling the temperature distribution of the mold. It has been validated that conformal cooling channel can get more uniform temperature distribution on the mold surface, shorten the cycle time, and improve the quality. The direct way of conformal cooling is using baffles in the cooling system [32]. Wang et al. [26,33] proposed a cooling channel design method based on Voronoi Diagram. Cooling time results of two different parts were decreased by 26% compared with the conventional channels. Li et al. [27] conducted a work about topological design method of cooling channel for injection molding. The governing equations and sensitivities in cooling analysis were solved by using boundary element method (BEM). The results emphasized that the optimized topological cooling channel can get better efficiency and uniformity in cooling process than the conventional cooling channel. Kitayama et al. [31] conducted the research on reducing warpage, cycle time and clamp force by using conformal cooling channel system. Three objectives were improved compared with conventional result, especially 43% warpage reduction and 47% cycle time reduction.

In this paper, we focused on how to minimize the warpage deformation and improve the appearance quality around the injecting gate of our product. The interesting and innovative point in this paper is that we employed the insert technology embedded in the runner to influence the physical fields of the polymer melts before it enters the cavity. Three different shapes of inserts, which are circular, rectangular and diamond-shaped, were chosen to study the efficiency of insert shape on improving the final appearance quality. Three geometrical parameters optimization problems were successfully solved by using kriging surrogate model and expected improvement-based parameter optimization method accordingly. Results showed that not all design schemes with inserts are better than the design without insert in the runner. However, over 75% cases during optimization history are better than the original design. It indicates that appearance quality can be improved if the proper designed insert is used. The optimum rectangular design is the best one in three different shapes, which can reduce 21.56% surface deformation compared with the case without insert.

## 2. Geometry and Material

**Figure 1:**
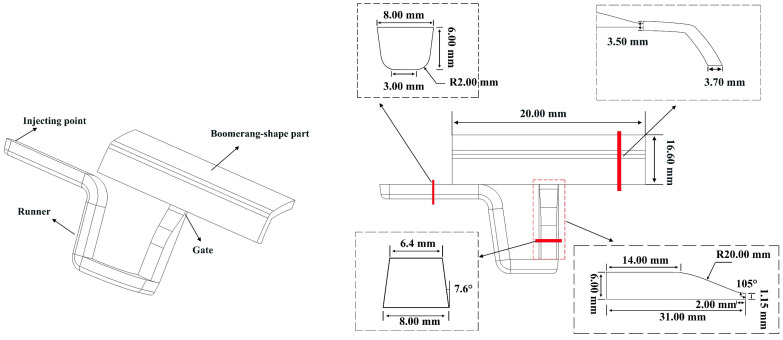
Geometry and the dimensions of the plastic part without insert close the gate.

The plastic part in this work is a typical standard part in auto industry. The total length and width are 20.00 mm and 16.60 mm, respectively. The cross section of this plastic part is uniform boomerang, of which thickness is 3.50 mm. The height and width of the injection gate are 1.15 mm and 8.00 mm, respectively. The cross section of the main runner is U-shape, while the cross section of the runner connected to the injection gate is trapezoid shape. Height of this trapezoidal section decreases from the end of U-shape runner to the injection gate in order to well connect to the gate. The draft angle in this part of runner is 7.6°. All these geometrical designs are taken into consideration for final ejection. Details of this L-shape plastic part and runner system are dimensioned in Figure 1.

**Figure 2:**
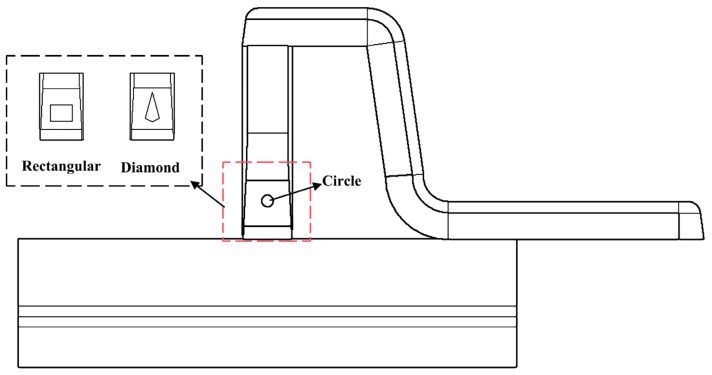
Location and geometrical shape of different inserts.

Inserts with different geometrical shape are illustrated as Figure 2, including circular, rectangular and diamond-shaped in this study. This insert was placed at the slope part of the runner which is close to the injection gate. Thus, it can absorb some heat from the polymer melts that pass by the insert, and also change the velocity field before running into the part cavity. The material state that surrounds the gate, like temperature, molecular orientation etc., will be changed accordingly. Eventually, the expected result is that the final appearance quality will be improved than the one without insert after optimizing the insert shape.

**Figure 3:**
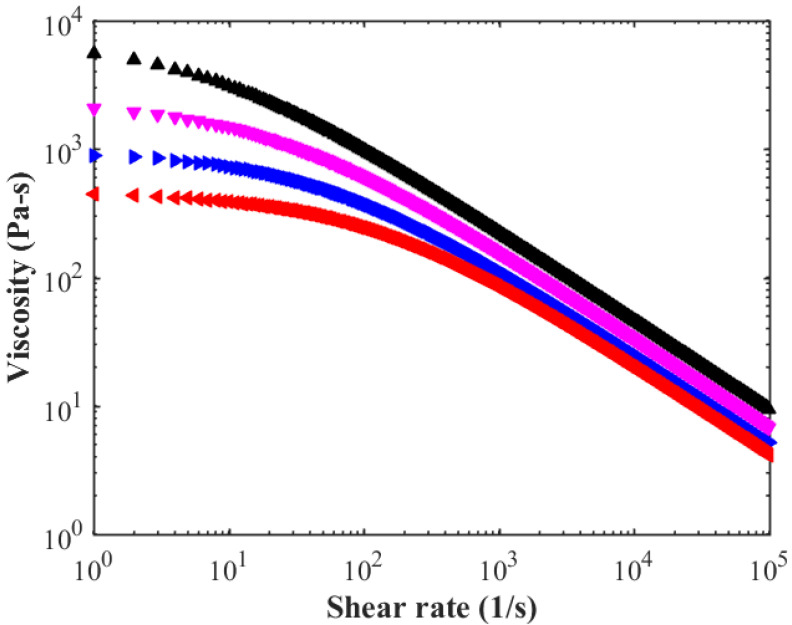
Viscosity curve of Cross-WLF model.

This standard plastic part is made of ABS granular material (INEOS ABS, Lustran PG 298). The density is 1.0560 g/cm^3^ for solid and 0.9486 g/cm^3^ for polymer melts. The widely used cross-WLF viscosity model is used to present the rheology behavior during injection molding. This famous model is expressed as:

(1) $$\eta =\frac{\eta_{0}}{1+{\left(\frac{\eta_{0}\dot{\gamma }}{\tau *}\right)}^{1-n}}$$

where

(2) $$\eta_{0}=D_{1}\exp\left[-\frac{A_{1}\left(T-T*\right)}{A_{2}+T-T*}\right]$$

(3) $$T*=D_{2}+D_{3}\cdot p$$

(4) $$A_{2}={\bar{A}}_{2}+D_{3}\cdot p$$

where τ*, *n*, *D*_1_, *D*_2_, *D*_3_, *A*_1_, ${\bar{A}}_{2}$ are material constants and listed in Table 1 for INEOS ABS, Lustran PG 298. $\dot{\gamma }$ is the shear rate in the polymer melts, and equals to $\sqrt{\frac{1}{2}\sum_{i=1}^{dim}{\dot{\gamma }}_{\mathrm{ij}}{\dot{\gamma }}_{\mathrm{ji}}}$ in which *dim* represents the dimension and ${\dot{\gamma }}_{\mathrm{ij}}$ is the rate of strain tensor in the polymer melts. The parameters are listed in Table 1. The corresponding viscosity curves can be plotted as Figure 3 based on these parameters, provided by Autodesk Moldflow database.

## 3. Simulation and Methods

### 3.1. Processing Parameters

**Figure 4:**
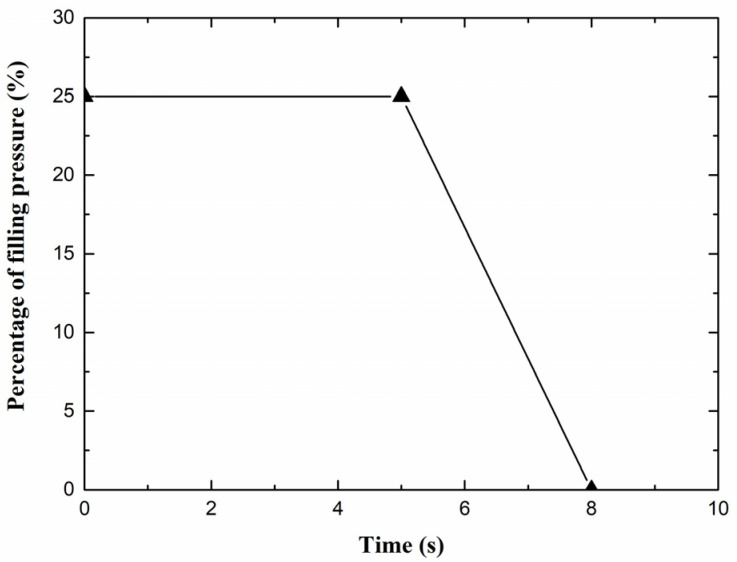
Packing process curve in this study.

The purpose of this work is to study the influence of the insert on the appearance quality in the area which is around the injection gate. Therefore, the processing parameters are kept the same during geometrical optimization process on the insert. The injection time and cooling time are 0.1 s and 20 s, respectively. The percentage of the filled volume, which is 98%, is employed for velocity/pressure switch condition. A right-angled trapezoid packing process is used for packing control. As Figure 4 shown, the relationship between percentage of filling pressure and time is used as the packing condition. Moreover, other process parameters are recommended and listed as Table 2 including the mold temperature, melt temperature, ejection temperature in cavity and mold open time.

### 3.2. Geometry Optimization of Insert

**Figure 5:**
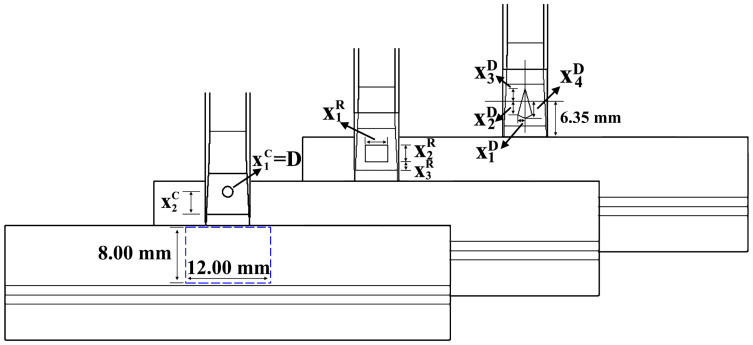
Geometrical optimization parameters of different shape inserts.

There are three types of insert in this study. Each type of insert has its own parameters to control the dimensions and the geometrical location in the running system. Figure 5 is the projection scheme of the part. For circular insert, diameter of the circle is the first parameter ($x_{1}^{c}$) to describe its size. Another parameter ($x_{2}^{c}$) is the distance between the center of the circle and the beginning line of the injection gate, not the distance between the center of the circle and the surface which is connected to the injection gate. The reasons are to keep the integrity of the gate, avoid weld line in the part and avoid blocking the entrance into the part cavity as much as we can. For rectangular shape, the first and second parameters ($x_{1}^{R}\;$ and $\;\;x_{2}^{R}$) are the width and height of the rectangular, respectively. The third one $x_{3}^{R}$ is the distance between the bottom side of the rectangular and the beginning line of the injection gate. If $x_{1}^{R}\;$ equals $x_{2}^{R}$, rectangular will yield into square shape. For diamond-shaped insert, the first parameter $x_{1}^{D}$ is the distance between the shoulder point of the diamond and the center line of the diamond. Other parameters are all based on one reference line, which is 6.35 mm to the surface of the part. This arrangement is to make optimization simple as much as possible, and avoid concave geometry, which is not diamond-shaped, although it can be realized by constraints. The second parameter $x_{2}^{D}$ is the distance between the shoulder point of the diamond and this reference line. The third one $x_{3}^{D}$ is the distance between the head point of the diamond and this reference line. The last parameter $x_{4}^{D}$ is the distance between the tail point of the diamond and this reference line. $x_{1}^{D}$, $x_{2}^{D}$ and $x_{3}^{D}$ are all below the reference line. Only $x_{4}^{D}$ is above the reference line. Two things should be noticed. One is that diamond will yield into triangle shape if two shoulder points and the head (tail) point are aligned horizontally. The other is that diamond-shaped insert will also yield into square shape insert in another point of view at certain conditions. It means that these four parameters can totally describe three geometries. All dimensions were parameterized in Pro-E software v5.0, and changed according to the new parameters which were obtained during optimization.

The deformation around the gate is usually not good enough to satisfy strict appearance requirement. Therefore, the objective in this paper is the surface deformation in y-axis in 8 mm × 12 mm region close to the gate, which has been identified by blue dash line in Figure 5. The deformation is obtained by the powerful commercial injection molding software Autodesk Moldflow insight 2018. All the models for simulation were well meshed by setting the element size automatically with our self-developed program to ensure the simulation precision [29].

The objective is an implicit function y(**x**) with respect to all the design parameters. Therefore, the optimization problem is basically written as:

(5) $$\left\{\begin{array}{l} Find:\;x^{l}\\ Min:\;\;y(x^{l})=d_{y}\left(x^{l}\right)\\ S.T.:\;x_{lb,i}^{l}\leq x_{i}^{l}\leq x_{ub,i}^{l},\;x_{i}^{l}\in x^{l},\;i=1,2,\ldots ,N^{l}\; \end{array}\right.$$

where *x^l^* represents the parameters of the shape *l*. *d_y_*(x) is the surface deformation in y-axis in the confined region in Figure 5. All surface nodes in this region will be considered as the target nodes. Warpage analysis was performed to obtain the deformation after ejection. The deformation of these nodes represents the smoothness of the surface. The smaller maximum deformation in this region is, the smoother the surface is, the higher the surface quality is. $x_{lb,i}^{l}$ and $x_{ub,i}^{l}$ are the lower limit of the *i*-th parameter for the *l*-th shape, which are tabulated in Table 3. *N^l^* is the parameter number of the *l*-th shape.

In this study, kriging surrogate model [4,29,34,35] is employed, which includes a polynomial part and a stochastic function as:

(6) $$y\left(x\right)=\sum_{i=1}^{m}\beta_{i}f_{i}\left(x\right)+\varepsilon \left(x\right)$$

where *β*_i_ and *f*_i_(**x**) are regression coefficient polynomial. *ε*(**x**) is a function which follows norm [0, σ^2^] distribution.

There are many expressions about kriging model. Herein, the ordinary kriging (OK) model is employed. In OK model, only the zero-order part is remained. Then, the prediction function and variance function at any design point x* is expressed as:

(7) $$\bar{y}\left(x*\right)=u+r^{T}\left(x*\right)\cdot R^{-1}\cdot \left(Y-u\cdot l\right)$$

(8) $${\tilde{s}}^{2}\left(x*\right)={\tilde{\sigma }}^{2}\left\{1+\frac{{\left[1-l^{T}R^{-1}r\left(x*\right)\right]}^{2}}{l^{T}R^{-1}l}-r^{T}R^{-1}r\left(x*\right)\right\}$$

where *u* = (**l**^T^**R**^−1^**l**)^−1^(**l**^T^**R**^−1^**Y**). The element in **l** equals one. ${\tilde{\sigma }}^{2}$ is the maximum likelihood of σ^2^. **Y** is the objective vector with respect to design sample matrix **X**. r*_i_* = ***R***(*λ*, x*, **x**_i_). *λ* is correlated coefficient. **x**_i_∈**X**. **R** is correlation function matrix. For Gaussian form, it is:

(9) $$R_{ij}\left(\lambda ,x_{i},x_{j}\right)=\prod_{k=1}^{m}\exp\left[-\lambda^{k}{\left(x_{i}^{k}-x_{j}^{k}\right)}^{2}\right]$$

Since the precision of surrogate model on real function depends on the number of interpolation samples and the corresponding distribution. Direct optimization about the surrogate model is not the best way. Therefore, some transforms for objective are employed to solve the optimization problem well. According to the characters of kriging surrogate model, the most efficient searching function is the expected improvement (EI) function [36], which reads as:

(10) $$EI=E[I\left(x\right)]=\tilde{s}\left(x\right)\cdot \phi [\frac{y_{\min}-\bar{y}\left(x\right)}{\tilde{s}\left(x\right)}]+[y_{\min}-\bar{y}\left(x\right)]\cdot \Phi [\frac{y_{\min}-\bar{y}\left(x\right)}{\tilde{s}\left(x\right)}]$$

where *I*(**x**) = *y*_min_ − *y*(**x**). *EI* is the expectation of *I*(**x**) in the positive region part. *y*_min_ is the minimum value in the current interpolation samples **Y**.

The lager *EI* is, the larger $\tilde{s}\left(x\right)$ is or the lower $\bar{y}\left(x\right)$ is than *y*_min_. It means that, the maximum of *EI* in the design domain represents the most uncertain point for prediction by kriging model or a new minimum point than the current one. If we minimize *EI* by optimization procedure, we can improve the precision of the model and find the final optimal result of the problem. Therefore, the optimization expression is transformed into Equation (11). In this paper, Sequential Quadratic Programming (SQP) is used to solve the optimization problem. All optimization works were coded and performed in MATLAB 2016. New dimensions will be updated in Pro-E software v5.0 during the optimization automatically.

(11) $$\left\{\begin{array}{l} Find:\;\;x^{l}\\ Min:\;\;\max\;EI\left(x^{l}\right)\\ S.T.:\;x_{lb,i}^{l}\leq x_{i}^{l}\leq x_{ub,i}^{l},\;x_{i}^{l}\in x^{l},\;i=1\ldots ,N^{l}\; \end{array}\right.$$

**Figure 6:**
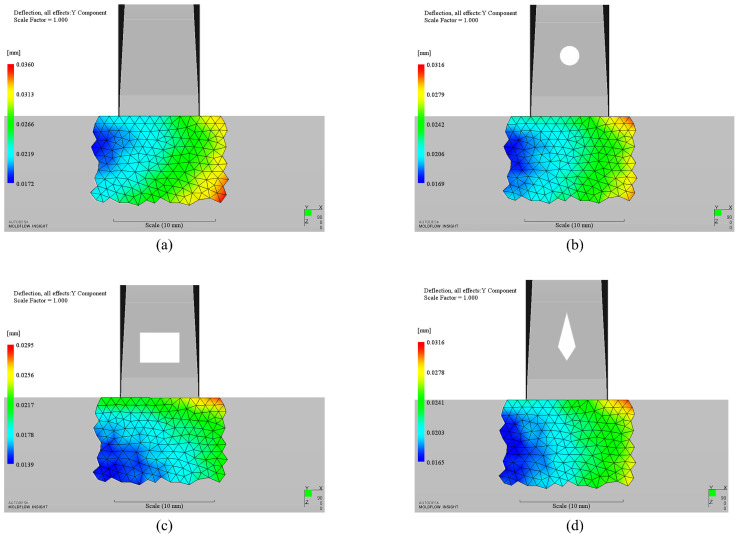
Deformation contour of optimization cases: (**a**). Without insert; (**b**). Circular insert (optimized); (**c**). Rectangular insert (optimized); (**d**). Diamond-shaped insert (optimized).

**Figure 7:**
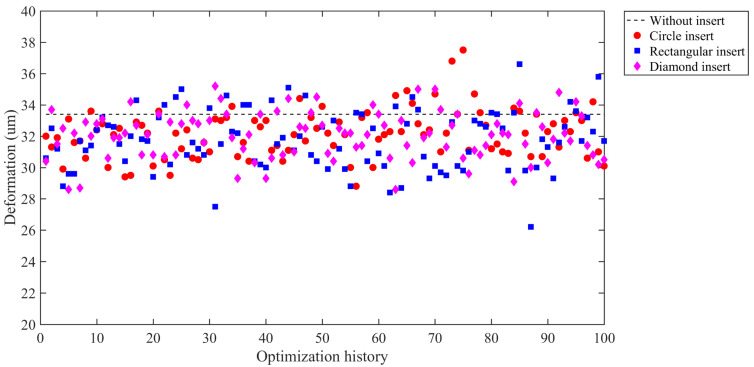
Appearance quality optimization history of different shape inserts.

The final optimization results are tabulated in Table 4. The maximum deformation in the concerned region of the product without any insert is 33.4 μm as shown in Figure 6a. The optimized maximum deformation results of all insert cases are 28.8 μm, 26.2 μm, 28.6 μm for circular, rectangular and diamond-shaped insert respectively (Figure 6b–d), which are all less than the original design case (without insert). Accordingly, the appearance quality is improved by 13.77%, 21.56%, 14.37%, respectively. The results revealed that appearance quality with optimized rectangular insert is the best among three cases. The most important thing is that, compared with the original design, the maximum deformation of each insert case moves from the key appearance surface to the side of the product. It is an attractive and good news that not only the appearance quality is improved, but also the maximum deformation happens at an unconsidered location. Additionally, surface deformations of most cases for each shape are lower than the original design in the optimization history (Figure 7), 80% for circular, 77% for rectangular and 78% for diamond-shaped, respectively. It indicates that appearance quality can be well improved by introducing insert in the runner system if the geometry of the insert is well designed.

There are two interesting things. One is that the projection areas of circular, rectangular and diamond inserts are 3.17, 12.28 and 4.22 mm^2^, and appearance quality is accordingly improved by 13.77%, 21.56%, 14.37% for each optimized case, respectively. It seems like that the smaller the area of the shape is, the less the appearance quality is improved among three optimized design schemes. The other is the center of circular, rectangular, and diamond-shaped inserts to the side of the part are 6.15 mm, 5.04 mm, and 5.14 mm (intersection point of diagonals), respectively. It also seems like that the closer to the side the insert is, the less the appearance quality is improved.

## 4. Discussion

**Figure 8:**
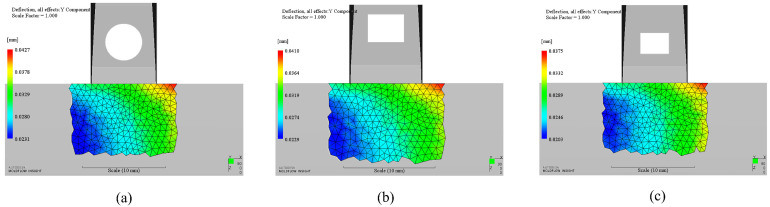
Deformation contour of supplementary cases: (**a**). Circular case located at the center of the optimized rectangular case with the same area as this rectangular; (**b**). Rectangular case with the same dimensions as the optimized rectangular case, but 1 mm farther; (**c**). Rectangular case located at the center of the optimized rectangular case, but 0.5 mm smaller in length and width.

There are three key factors to affect the final appearance quality. The first one is the geometrical shape. The second one is the geometrical location of the insert. The last one is the dimensions of the geometrical shape. However, the final appearance quality is determined by all factors. For instance, we choose another three supplement design schemes to explain the influence of these three factors on the surface deformation. The first one is a circular case, which locates at the center of the optimized rectangular case with the same parameter as this rectangular. It was used to demonstrate the influence about shape on appearance quality. The second one is a rectangular case with the same dimensions as the optimized rectangular case but is 1 mm farther than the optimized rectangular case to the side. It was used to demonstrate the influence about location on appearance quality. The third one is also a rectangular case, which locates at the center of the optimized rectangular case while the sides are 0.5 mm smaller than the optimized rectangular case. It was used to demonstrate the influence about dimensions on appearance quality. Similarly, the maximum deformation of each case locates on the side of the product. However, the maximum deformation results of these three cases are 39.9 μm, 38.3 μm and 35.0 μm as shown in Figure 8, respectively. The deformations are all higher than the optimization result of rectangular insert. It indicates that the best result must come from the optimum combination of geometrical shape, location, and dimensions of the insert when all the processing parameters are the same.

**Figure 9:**
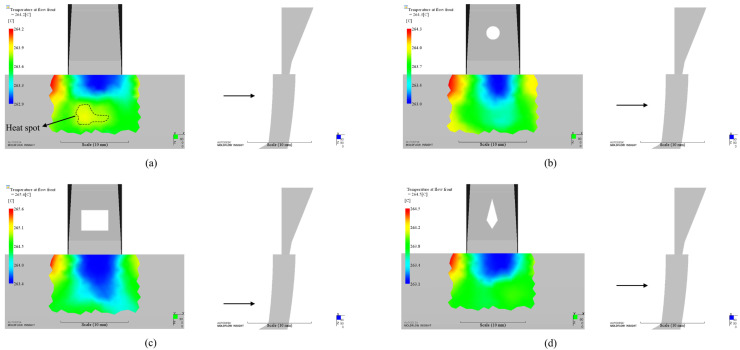
Temperature distribution at flow front and the ending location of lower temperature: (**a**). Without insert; (**b**). Circular insert (optimized); (**c**). Rectangular insert (optimized); (**d**). Diamond insert (optimized).

The lateral view in Figure 9 shows the difference about the ending location of lower temperature. Compared with other cases with inserts, one phenomenon is that there is a heat spot on the part which cannot be neglected in the original design case. The temperature of this heat spot is higher than the surrounding material. This part of material will take more time to cool down. It will stretch the surrounding material and result in the final surface sink mark due to this ununiform temperature distribution. By contrast, we did not observe such heat spot in any case with insert. It indicates that insert can eliminate it efficiently. Another phenomenon is that lower temperature region of any case with insert distributes more extensively from the side of the part than the case without insert.

**Figure 10:**
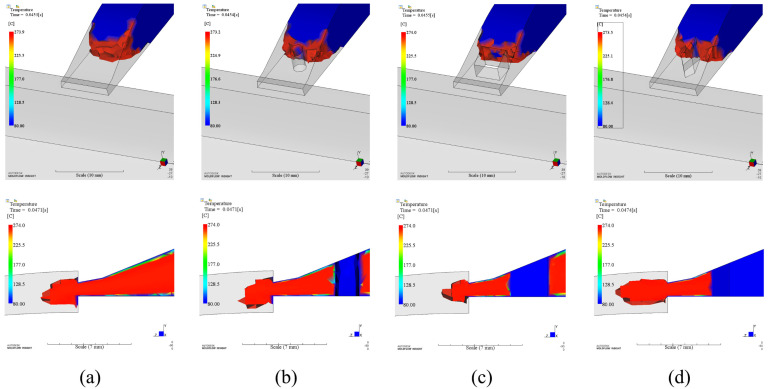
Temperature of melts before entering the cavity: (**a**). Without insert; (**b**). Circular insert; (**c**). Rectangular insert; (**d**). Diamond insert.

**Figure 11:**
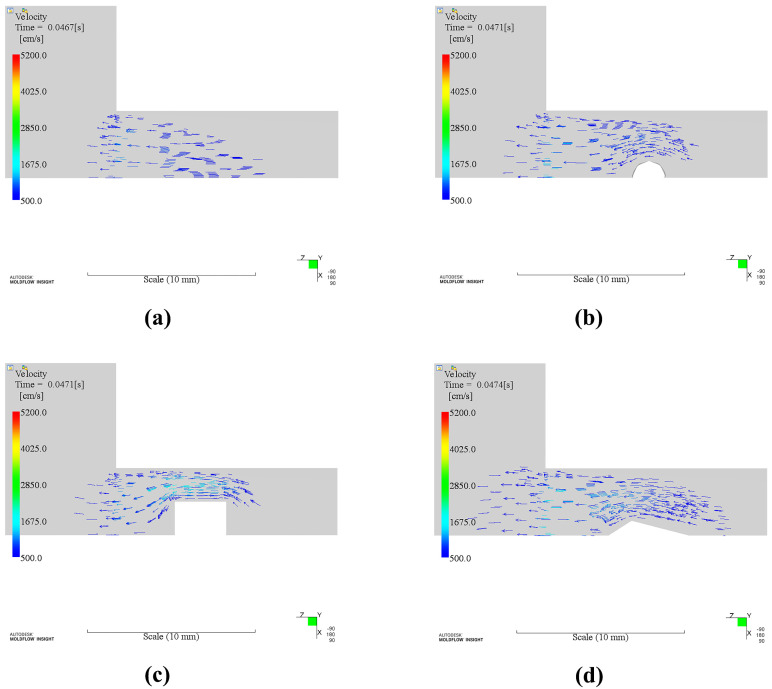
Velocity fields of different cases: (**a**). Without insert; (**b**). Circular insert; (**c**). Rectangular insert; (**d**). Diamond insert.

In order to explain the reason why appearance quality is improved by using inserts around the gate, we analyzed the physical fields change for all cases. The insert was embedded in the runner before the injection gate. The polymer melts will meet the insert before it enters the mold cavity (Figure 10). Any melts that passed by the insert will be split into two flows (Figure 10 before). Part heat of that melts will be taken away by the insert. The temperature of that melts should be reduced. However, velocity gradient of the melts increased since the cross-section area decreased due to the insert (Figure 11). The temperature of the skin layer was increased by strong shear effect with the help of inserts (Figure 10 after). This effect was strongest for rectangular insert than others in terms of the balance between the magnitude of shear force and heat absorption.

**Figure 12:**
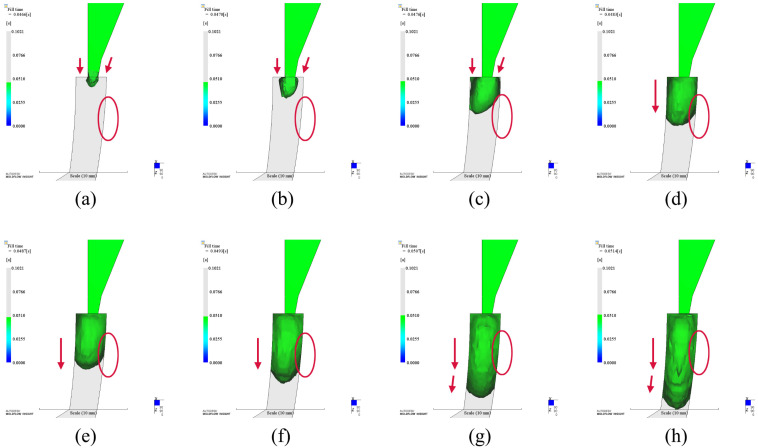
Flow progress of the melts after entering the cavity in the case of without insert.

**Figure 13:**
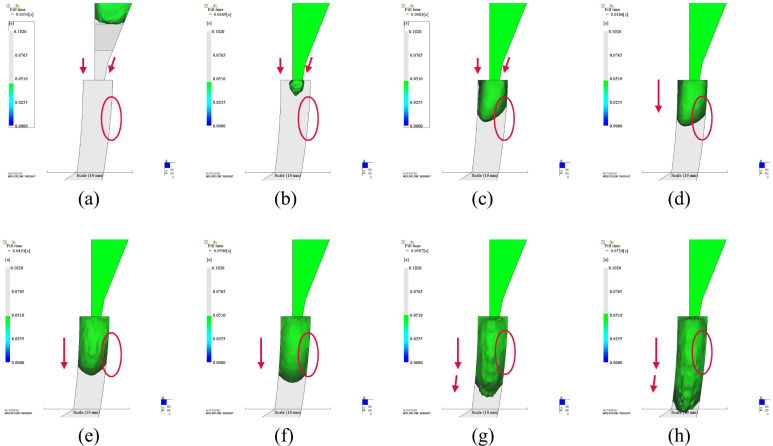
Flow progress of the melts after entering the cavity in the case of rectangular insert.

If we tracked the progress of the flow, we could observe that the polymer melts reached the bottom surface first after the melts ran into the cavity for the original design (Figure 12a-c) since the injection gate was a contraction type (Figure 1). It was not symmetric when the melts reached both main surfaces in Figure 12c. The contact area between plastic and mold on the top surface was smaller than that on the bottom surface at this time point. Then, flow direction of the front melts was changed according to the curved surface of the product after the melts reached both main surfaces (Figure 12d–h). Subsequently, the hotter melts in the core layer moved toward the top surface of the product. Therefore, it can transfer more heat from the hotter melts to the mold surface, and results in heat spot in the first phenomenon. After the polymer melts entered the cavity, the melts also reached the bottom surface first for rectangular case (Figure 13a–c). Because there was a lag for reaching the mold surface compared with the bottom side, the heat spot was eliminated due to much uniform temperature inside the core material for all shapes of insert. For the same reason, the lower temperature regions for three shapes were extended. The extended level was related to the contact area between the melts and the insert. The larger the contact area is, the larger the lower temperature region is. For the current optimization results, the circular insert has the smallest contact area while the rectangular insert has the largest one. Consequently, the area of lower temperature region in circular insert case is the smallest while it is largest in rectangular case.

**Figure 14:**
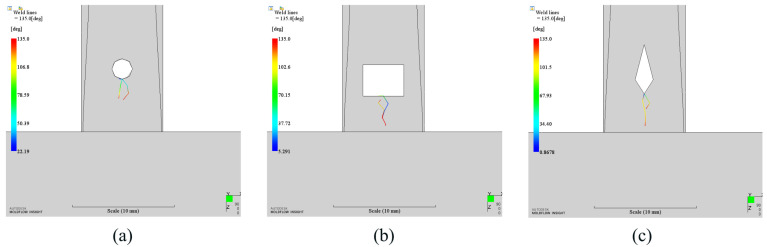
Weld lines results of different shapes. (**a**). Circular insert (optimized); (**b**). Rectangular insert (optimized); (**c**). Diamond insert (optimized).

Any melts will be split into two parts after passing by the insert. When they met again, it will introduce weld lines. Weld line is a fatal defect for the product which needs high appearance requirement. We also investigated the weld lines of the optimization results. As shown in Figure 14, there exist weld lines around the insert in all cases. The length of weld line in diamond case is the longest, while it is the shortest in circular case. However, all weld lines end before the auto part. It will not affect the appearance of the auto part and meets the requirement of appearance.

Overall, the effect of insert on improving the appearance quality around the gate is apparent. The key is how to well design the geometry of the insert including the shape, dimension, and location.

## 5. Conclusions

Appearance quality is important for many injection-molded products. Shrinkage and warpage are main defects which can cause bad appearance around the injection gate. Based on the most efficient optimization method, geometrical design problem of the gate insert was solved to improve the surface deformation of the standard auto part. Results of three different shape inserts present better appearance quality than the original design which didn’t use any insert around the gate. However, it also indicates that geometry of the insert should be well designed, or it will induce worse appearance quality than the original design. The insert can absorb part heat of the melts that flow around the insert, and affect the complicated thermo-mechanical history. Weld lines results showed that the final design cannot introduce any weld line into the auto part. This work suggest another way to improve the quality of injection molded product, especially for the products that require high appearance index.

## Figures and Tables

**Table 1:** Parameters of Cross-WLF viscosity model for ABS.

Parameter	τ* (Pa)	*n*	*D*_1_ (Pa-s)	*D*_2_ (K)	*D*_3_ (K/Pa)	*A*_1_	${\bar{A}}_{2}$ (K)
Value	52,540	0.3046	2.3 × 10^14^	373.15	0	34.66	51.6

**Table 2:** Recommended process parameters for ABS (Lustran PG 298).

Process Parameter	Magnitude
Mold temperature (°C)	80
Melt temperature (°C)	260
Ejection temperature in cavity (°C)	93
Mold open time (s)	5

**Table 3:** Boundaries for each shape.

Parameter	$x_{1}^{C}$	$x_{2}^{C}$	$x_{1}^{R}$	$x_{2}^{R}$	$x_{3}^{R}$	$x_{1}^{D}$	$x_{2}^{D}$	$x_{3}^{D}$	$x_{4}^{D}$
*x_lb_*	1.00	3.00	1.00	1.00	1.00	0.50	−2.00	2.35	2.00
*x_ub_*	4.00	5.50	4.00	4.00	3.50	2.00	2.00	4.25	4.00

**Table 4:** Optimized results of different shape inserts.

Shape	$x_{1}$ (mm)	$x_{2}$ (mm)	$x_{3}$ (mm)	$x_{4}$ (mm)	Deformation (μm)	Efficiency (%)
Without insert	-	-	-	-	33.4	-
Circular	2.01	4.15	-	-	28.8	13.77
Rectangular	4.00	3.07	1.50	-	26.2	21.56
Diamond-shaped	0.88	1.21	2.59	2.21	28.6	14.37

## Data Availability

Not applicable.
